# Gut microbiota dysbiosis and hepatic inflammation in morphine dependence and withdrawal: insights from a rat model

**DOI:** 10.1186/s12876-026-04669-w

**Published:** 2026-02-09

**Authors:** Shirin Yousefi, Mahsa Sadeghi-Adl, Samira Tarashi, Seyed Davar Siadat

**Affiliations:** 1 Advanced-Sciences and Technology Faculty, Tehran Medical Branch, Islamic Azad University, Tehran, Iran; 2 Iranian National Center for Addiction Studies, Tehran University of Medical Sciences, Tehran, Iran; 3 Microbiology Research Center (MRC), Pasteur Institute of Iran, No. 358, 12th Farvardin Ave, Jomhhoori St, Tehran, Iran; 4 Department of Mycobacteriology and Pulmonary Research, Pasteur Institute of Iran, Tehran, Iran

**Keywords:** Opioid abuse, Morphine dependence, Gut-liver axis, Inflammation, Microbiota

## Abstract

**Background:**

Opioid dependence, particularly morphine, has been linked to gut microbiota dysbiosis and systemic inflammation, yet the interplay between gut microbial alterations and hepatic inflammatory responses remains poorly understood.

**Methods:**

Fifty male Wistar rats were separated into two groups, one received escalating morphine doses (5 to 30 mg/kg over 10 days), while the other acted as a saline control. Fecal samples were collected at baseline, on days 5 and 10 of treatment, and after a 10-day withdrawal. DNA was extracted for qPCR analysis of *Lactobacillus*, *Bifidobacterium*, *Clostridium*, *Bacteroides*, and *Faecalibacterium*. Liver tissues were examined for inflammatory markers (*TNF-α*, *IFN-γ*, *IL-6*, *NF-κB*) using RT-qPCR after treatment and withdrawal.

**Results:**

A significant decline in *Lactobacillus* (*P* = 0.011) and *Bifidobacterium* (*P* = 0.003) following morphine treatment, with partial recovery observed after withdrawal (*P* = 0.014; *P* = 0.0009), yet levels remained below baseline. Conversely, *Clostridium* levels increased significantly during treatment (*P* = 0.0001), persisting at elevated levels post-withdrawal (*P* = 0.0001). *Bacteroides* and *Faecalibacterium* also exhibited decreased abundances during morphine treatment (*P* > 0.05; *P* = 0.00009), with limited recovery thereafter (*P* > 0.05; *P* = 0.00008). Hepatic analysis revealed elevated levels of *TNF-α* (*P* < 0.0001), *IL-6* (*P =* 0.005), and *NF-κB* (*P =* 0.41), alongside a significant reduction in *IFN-γ* (*P* < 0.001) expression in the morphine group compared to controls. After withdrawal, *TNF-α* (*P* < 0.01) and *IFN-γ* (*P* = 0.004) levels decreased, while *NF-κB* (*P* = 0.03) and *IL-6* (*P =* 0.4(remained elevated, indicating persistent inflammatory responses.

**Conclusion:**

Morphine causes lasting gut dysbiosis and liver inflammation, indicating disruption of the gut-liver axis in opioid dependence. These results emphasize morphine’s impact on gut microbiota and liver health, suggesting significant long-term effects of opioid use. Targeting microbiota modulation and anti-inflammatory approaches may offer therapeutic options for opioid-related conditions.

## Introduction

Substance use disorders, including opioid dependence, represent a significant global public health challenge and are among the leading causes of preventable mortality worldwide [1]. Morphine, a potent opioid analgesic derived from the opium poppy, is essential for managing severe pain but carries a high risk of abuse and addiction [2]. Its activation of central nervous system receptors induces not only analgesia and euphoria but also leads rapidly to physical and psychological dependence. This dependence syndrome is characterized by a compulsive drive to use the substance, loss of control over intake, and continued use despite harmful consequences [3]. The development of effective treatment strategies remains difficult, highlighting the need for a deeper understanding of the underlying pathophysiology [4]. The urgent need for new and effective treatments for morphine addiction is clear, yet developing successful addiction management strategies presents significant challenges [5]. Regarding the mechanisms, opioids exert their effects through various opioid receptors, which are predominantly expressed in the gastrointestinal nervous system. Four primary types of opioid receptors have been identified, including mu (MOP), delta (DOP), kappa (KOP), and nociceptin/orphanin opioid receptors (NOP) [6]. As an exogenous opioid ligand, morphine activates these receptors, influencing not only pain perception but also the functioning of the digestive and immune systems [7].

Currently, it is widely recognized that the gastrointestinal microbiota is essential for maintaining host health. It does this by regulating the function of the digestive barrier, generating energy from dietary inputs, preventing the colonization of pathogens, modulating metabolic processes, and balancing immune responses [8]. Furthermore, the gut microbiota is intricately linked to liver function, with interactions between these two systems mediated by immune system factors [9]. Dysbiosis, or imbalances in gut microbiota composition, can disrupt host functions and contribute to various disorders [10]. The gut-liver axis has garnered significant attention as a vital area of research, particularly in understanding the intricate relationships between the gastrointestinal system and liver function in the context of drug dependence and addiction [11]. Evidence suggests that morphine induces notable alterations in gut microbiota and liver metabolism, potentially exacerbating its addictive properties and withdrawal symptoms [12–14]. Recent studies indicate that inflammation plays a critical role in the development of morphine dependence, with the gut-liver axis acting as a potential mediator of this inflammatory response [15]. The bidirectional communication between the gut and liver can significantly influence systemic inflammation, thereby affecting dysfunctions associated with addiction [16]. This study aims to investigate the significance of the gut-liver axis and its inflammatory pathways in a morphine dependence rat model over time. By examining changes in gut microbiota, liver function, and inflammatory markers at various stages, it is hoped to illuminate the underlying mechanisms that contribute to morphine dependence and withdrawal. Insights gained from this research may pave the way for novel therapeutic strategies to manage opioid addiction and its associated complications.

## Materials and methods

### Experimental animals

Fifty adult male Wistar rats, each weighing between 220 and 250 g, were sourced from the Pasteur Institute of Iran. Up to four rats were housed in each Plexiglas cage, with food and tap water provided ad libitum. The housing facilities were maintained under a 12-hour light-dark cycle (with lights on from 07:00 to 19:00), a constant temperature of 22 ± 2 °C, and humidity levels of 55 ± 5%. The study’s protocols and procedures received approval from the animal ethics committee of the Research and Ethics Committee of the Islamic Azad University of Medical Sciences in Tehran, Iran (IR.IAU.PS.REC.1403.279). All procedures adhered to the guidelines established by the National Institutes of Health Guide for the Care and Use of Laboratory Animals.

### Animal treatment

**Fig. 1:**
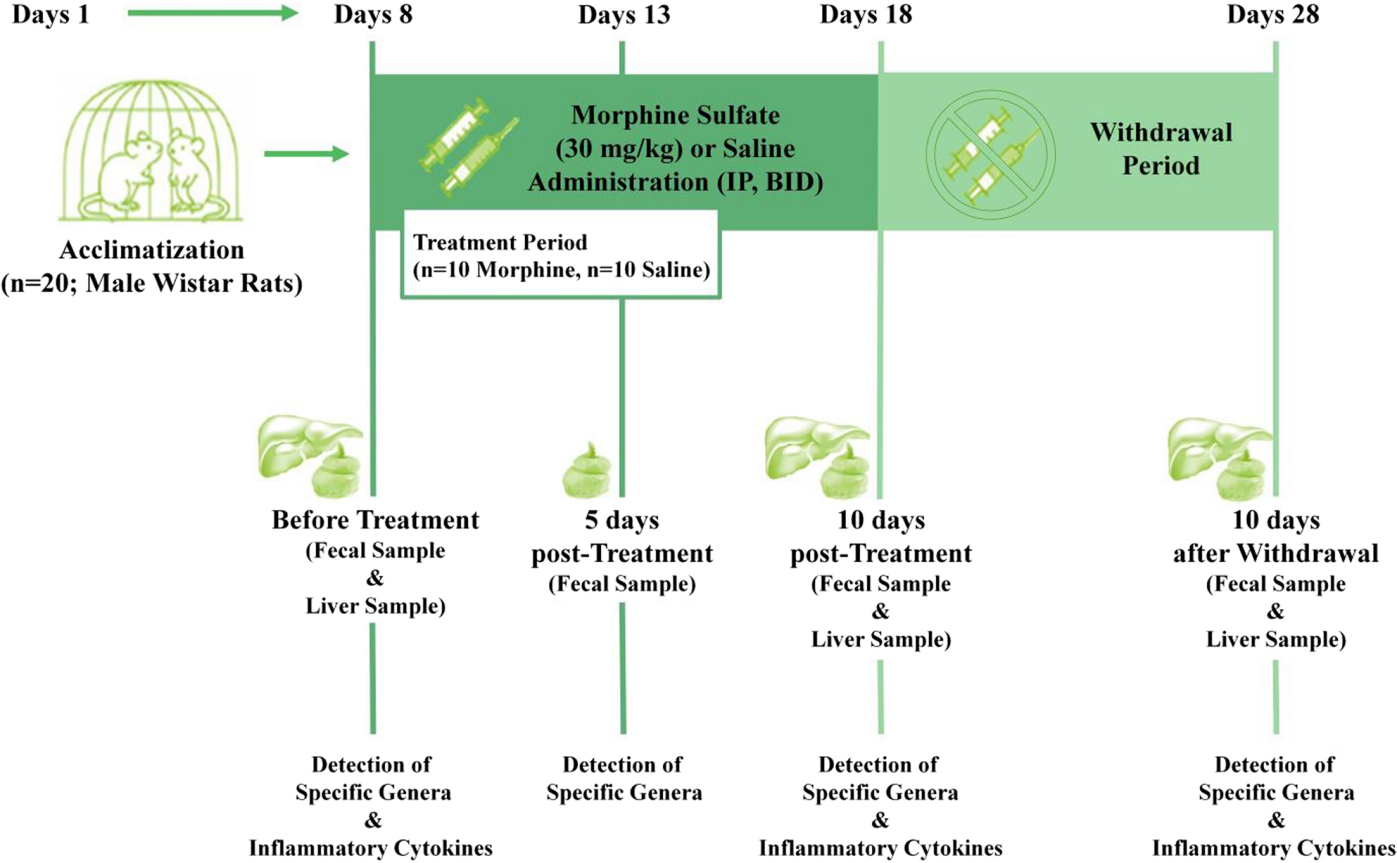
Schematic representation of the experimental protocol. Male Wistar rats (*n* = 20) underwent 7 days of acclimation. Morphine sulfate (30 mg/kg, equivalent to 26.4 mg/kg morphine base) or saline was administered intraperitoneally twice daily from Day 8 to Day 18. Fecal samples were collected on Days 0, 5, and 10 post-treatment and also 10 days after withdrawal period for detection of specific bacterial genera. Liver samples were also harvested on Days 0 and 10 post-treatment and 10 days after withdrawal period for detection of inflammatory cytokines through molecular analysis. IP: intra-peritoneal; BID: twice daily

After a week of acclimatization, the rats were randomly divided into two groups (*n* = 10 per group). One group received a single intraperitoneal injection of morphine, while the control group was given saline. Following this treatment, the rats underwent a 10-day withdrawal period. Fecal and liver samples were categorized and collected. Morphine sulfate was sourced from Temad Company (Tehran, Iran) and dissolved in sterile 0.9% saline just prior to administration, at a volume of 0.1 ml per 100 g of body weight. The morphine dosage was gradually increased from 5 to 30 mg/kg over the 10-day period, as per previously established methods [17] to model the development of dependence, a process often studied using this repeated-exposure paradigm in rodents. Specifically, injections were administered twice daily at 8:00 a.m. and 6:00 p.m., with the following dosing schedule: first two days (5 mg/kg), second two days (10 mg/kg), third day (15 mg/kg), and fourth and fifth days (25 and 30 mg/kg only at 8:00 a.m.). Control animals received saline injections at the same intervals. An experimental diagram is provided in Fig. 1.

### Anesthesia and euthanasia

In our study, we used isoflurane as the anesthetic agent. Isoflurane is a potent inhalational anesthetic commonly used in laboratory animal procedures due to its rapid onset and smooth induction of anesthesia. Isoflurane was administered at a concentration of 1–2% in a flow of oxygen. The rats were placed in an induction chamber where the isoflurane was delivered through a vaporizer, allowing for a consistent and controlled concentration of the anesthetic gas. This method ensures that the rats became unconscious quickly and remained pain-free throughout the experimental interventions. After confirming that the rats were fully anesthetized, euthanasia was conducted via decapitation. This method was chosen for its speed and effectiveness, minimizing any distress or discomfort. Euthanasia was performed only after verifying that the rats exhibited a lack of reflexes (such as response to toe pinch) and were completely unconscious under isoflurane anesthesia.

### Fecal sample collection and DNA extraction

Fecal samples were collected in 1.7 ml RNase/DNase-free tubes (Catalog #: C-2170, Denville Scientific, Holliston, MA, USA) at various time points: before morphine treatment, five and ten days post-treatment, and 10 days after withdrawal. Samples from the control group were collected similarly. Immediately frozen on dry ice, the fecal samples were stored at − 80 °C. DNA extraction was performed using the QIAamp DNA Mini Kit (Qiagen, Valencia, CA, USA), following the manufacturer’s protocol with 0.2 g of fecal material. The quality and concentration of the extracted DNA were assessed using gel electrophoresis and a Nanodrop spectrophotometer (ND-1000; Nanodrop Technologies). All DNA samples were stored at − 80 °C until further amplification.

### Quantitative real-time PCR amplification for detection of specific genera

Quantitative real-time PCR was employed to quantify bacterial genomic abundances in fecal samples at 0, 5, and 10 days post-morphine treatment, as well as 10 days after withdrawal. Based on microbiota databases like Disbiome [18] and previous studies, the genera selected for analysis included *Lactobacillus*, *Bifidobacterium*, *Bacteroides*, *Faecalibacterium*, and *Clostridium*. Specific primer amplification was normalized to the 16 S rRNA gene, with nucleotide BLAST used to confirm primer specificity. PCR reactions were conducted in duplicate using the Roche LightCycler^®^ 96 system (Roche, Switzerland). Each 20-µl reaction contained distilled water, SYBR Green master mix (Takara, Japan), DNA template, and forward and reverse primers. The primer sequences used for quantitative RT-PCR reaction are presented in Table 1. The amplification process consisted of an initial heating step at 95 °C for 1 min, followed by 40 cycles of denaturation, annealing, and extension. A melting curve analysis was performed post-amplification. To calculate bacterial loads, serial dilutions of DNA from standard strains of *Escherichia coli* were prepared, and DNA concentrations for each bacterium in fecal samples were derived from the standard curve.

**Table 1:** 16SrRNA gene primer sequences used for quantitative RT-PCR reaction

Bacteria	Primer Sequence	Tm (°C)	Amplicon size (bp)	Reference
***Escherichia***	F-CATTGACGTTACCCGCAGAAGAAGC R-CTCTACGAGACTCAAGCTTGC	55	350	[55]
***Lactobacillus***	F-AGCAGTAGGGAATCTTCCA R-CACCGCTACACATGGAG	58	250	[56]
***Bifidobacterium***	F-TCGCGTCYGGTGTGAAAG R-CCACATCCAGCRTCCAC	58	400	[57]
***Bacteroides***	F-CTGAACCAGCCAAGTAGCG R-CCGCAAACTTTCACAACTGACTTA	56	450	[58]
***Faecalibacterium***	F-GGAGGAAGAAGGTCTTCGG R-AATTCCGCCTACCTCTGCACT	55	250	[55]
***Clostridium***	F-GCACAAGCAGTGGAGT R-AACTGTTTTGCCTCCTTC	58	465	[59]

### Liver sample collection and RNA extraction

To analyze levels of inflammatory cytokines (*TNF-α*, *IL-6*, *IFN-γ*, and *NF-κB*), anesthetized rats were decapitated, and liver samples were collected immediately. These samples were taken 10 days post-morphine treatment and 10 days post-withdrawal, with control samples collected similarly. The liver samples were frozen on dry ice and stored at − 80 °C. RNA extraction was performed using the Precellys 24 homogenizer and Trizol, following the manufacturer’s instructions. RNA quantity and quality were verified through agarose gel electrophoresis and a NanoDrop spectrophotometer (ND-1000, THERMO SCIENTIFIC, USA). Subsequently, a reverse transcription (RT) reaction was conducted to synthesize complementary DNA (cDNA) using total RNA, an oligo(dT) primer, and ReverTra Ace (Toyobo, Osaka, Japan).

### Relative real-time PCR amplification for detection of inflammatory cytokines

Messenger RNA levels of *TNF-α*,* IL-6*, *IFN-γ*, and *NF-κB* were analyzed using relative real-time PCR. The qRT-PCR was performed on a Rotor-Gene^®^ Q (Qiagen, Germany) real-time PCR system with BioFACT™ 2X Real-Time PCR Master Mix (BIOFACT, South Korea). *GAPDH* served as the reference gene. All primers and probes were designed using Oligo7 Primer Analysis Software version 7.60 (Molecular Biology Insights, Inc., Colorado Springs, CO) and are detailed in Table 2.

**Table 2:** primer sequences of inflammatory cytokines used for relative real-time PCR reaction

	Gene	Primer Sequence	Product Length (bp)
Cytokine genes	TNF-α	F-AGCCCATGTTGTAGCAAACCR-TGAGGTACAGGCCCTCTGAT	55
	IL-6	F-TGCCAAGGAGTGCTAAAG R-CTCCACAACCCTCTGCAC	197
	IFN-γ	F-AGACAGCCACTCACCTCTTCAG R-TTCTGCCAGTGCCTCTTTGCTG	132
	NF-κB	F-GTCTCCTCTGACTTCAACAGCG R-ACCACCCTGTTGCTGTAGCCAA	58
Housekeeping gene	gapdh	F-GGTCTCCTCTGACTTCAACA R-AGCCAAATTCGTTGTCATAC	183

### Data analysis

To measure microbiota load in fecal samples, a series of standard dilutions was prepared for each real-time PCR run. The threshold cycle (Ct) was determined when sample fluorescence met a preset threshold and was referenced to the standard curve. Samples were loaded in duplicate, and the mean values were used for analysis. Data were analyzed using SPSS software version 26, and GraphPad Prism software (v.6, GraphPad Software Inc.) was used for plotting. The normality of distribution was assessed using the Shapiro-Wilk test. For comparisons of microbial abundance and cytokine expression across multiple time points within the same group (e.g., morphine group at Day 0, 5, 10, and withdrawal), one-way repeated measures ANOVA was used, followed by Tukey’s post-hoc test for pairwise comparisons. For comparisons between the morphine and saline control groups at each specific time point, an unpaired two-tailed Student’s t-test (for normally distributed data) or Mann-Whitney U test (for non-normal data) was applied. Results are presented as mean ± standard error of the mean (SEM), with a *P*-value of less than 0.05 considered statistically significant.

### Result

### Morphine treatment leads to variation in specific genera of the gut microbiota

**Fig. 2:**
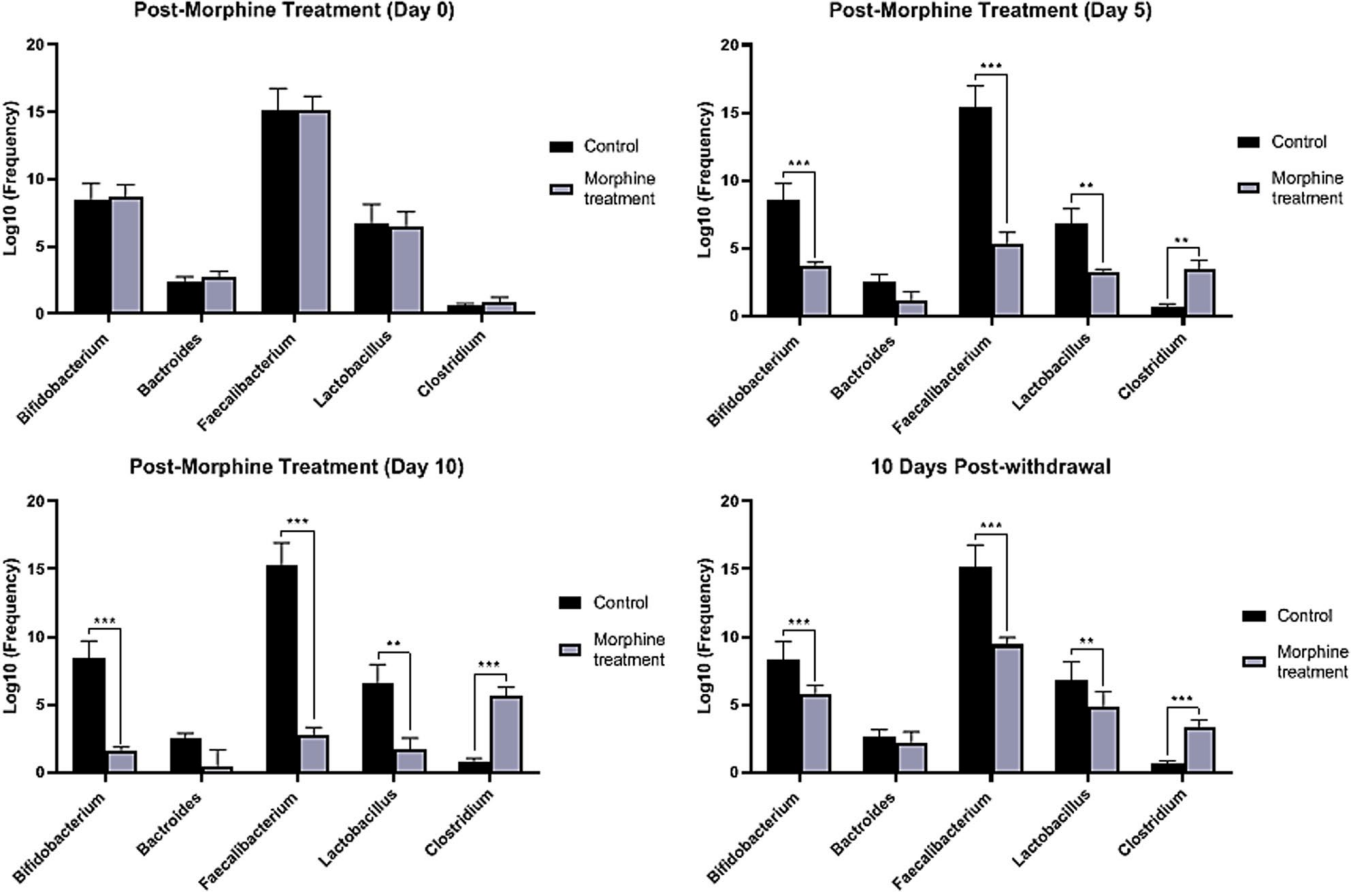
Comparison of abundance of bacteria between morphine-treated cases at different timelines and controls

To examine the relationship between changes in the genus level of gastrointestinal microbiota and morphine treatment over various timelines following treatment and withdrawal, we performed expression profiling of specific 16 S rRNA genes associated with selected genera in the gut microbiota using quantitative real-time PCR. Comparison of abundance of bacteria between morphine-treated cases at different timelines and controls are illustrated in Fig. 2. Prior to morphine treatment (Day 0), *Lactobacillus* (*P* > 0.05) and *Bifidobacterium* (*P* > 0.05) were present at stable levels. However, following 5 (*P* = 0.011; *P* = 0.003) and 10 days (*P* = 0.014; *P* = 0.0009) of morphine treatment, both genera exhibited a significant decline. After a 10-day withdrawal period, some recovery of *Lactobacillus* (*P* = 0.014) and *Bifidobacterium* (*P* = 0.0009) was observed, yet their levels remained below baseline. In contrast, *Clostridium* levels were low on Day 0 (*P* > 0.05) but increased significantly following morphine treatment (Day 5 (*P* = 0.005) and 10 (*P* = 0.0001)). Although *Clostridium* levels decreased after 10 days of withdrawal (*P* = 0.0001), they remained elevated compared to baseline. *Bacteroides* and *Faecalibacterium* demonstrated distinct responses to morphine. *Bacteroides*, which was abundant on Day 0 (*P* > 0.05), showed a gradual decline during morphine treatment, with a significant reduction after 5 (*P* > 0.05) and 10 days (*P* > 0.05). Despite partial recovery after 10 days of withdrawal (*P* > 0.05), *Bacteroides* levels remained below baseline. *Faecalibacterium* also exhibited severe depletion during morphine treatment (0-day, 0, *P* > 0.05; 5-day, *P* = 0.0005; 10-day, *P* = 0.00009), with limited recovery post-withdrawal (*P* = 0.00009). The reduction in *Faecalibacterium* levels after 10 days of withdrawal sustained.

Fecal analysis of PBS-treated control animals showed consistent microbial composition throughout the study period. The relative abundances of major gut bacteria-including *Lactobacillus*, *Bifidobacterium*, *Bacteroides*, *Faecalibacterium*, and *Clostridium*- remained stable (*P* > 0.05) across all time points (baseline, days 5 and 10 of treatment, and 10 days post-withdrawal). These finding confirms that PBS administration does not disrupt microbial composition over timelines exposure (Table 3).

**Table 3:** Comparison of abundance (Mean ± SEM) of bacteria between morphine-treated cases at different timelines and controls*

Bacterial Genera	Group	Day 0 Post-Morphine treatment (Mean ± SEM)	Day 5 Post-Morphine treatment (Mean ± SEM)	Day 10 Post-Morphine treatment (Mean ± SEM)	Withdrawal (Mean ± SEM)
***Bifidobacterium***	**Control**	8.432 ± 1.23	8.58 ± 1.18	8.39 ± 1.30	8.37 ± 1.26
	**Morphine treated**	8.648 ± 0.95	3.67 ± 0.29	1.53 ± 0.32	5.81 ± 0.59
***Bactroides***	**Control**	2.44 ± 0.31	2.54 ± 0.53	2.52 ± 0.37	2.68 ± 0.46
	**Morphine treated**	2.68 ± 0.46	1.08 ± 0.70	0.48 ± 1.18	2.22 ± 0.76
***Faecalibacterium***	**Control**	15.10 ± 1.62	15.38 ± 1.59	15.28 ± 1.66	15.16 ± 1.63
	**Morphine treated**	15.12 ± 1.04	5.36 ± 0.81	2.78 ± 0.54	9.43 ± 0.52
***Clostridium***	**Control**	0.61 ± 0.16	0.62 ± 0.26	0.75 ± 0.26	0.66 ± 0.14
	**Morphine treated**	0.93 ± 0.31	3.41 ± 0.68	5.61 ± 0.67	3.32 ± 0.53
***Lactobacillus***	**Control**	6.74 ± 1.39	6.82 ± 1.087	6.61 ± 1.36	6.76 ± 1.39
	**Morphine treated**	6.51 ± 1.07	3.25 ± 0.17	1.76 ± 0.77	4.83 ± 1.13

*Data are presented as Mean ± SEM

**P-value < 0.05 was considered statistically significant

### Morphine treatment alters the expression of hepatic inflammatory mediators

**Fig. 3:**
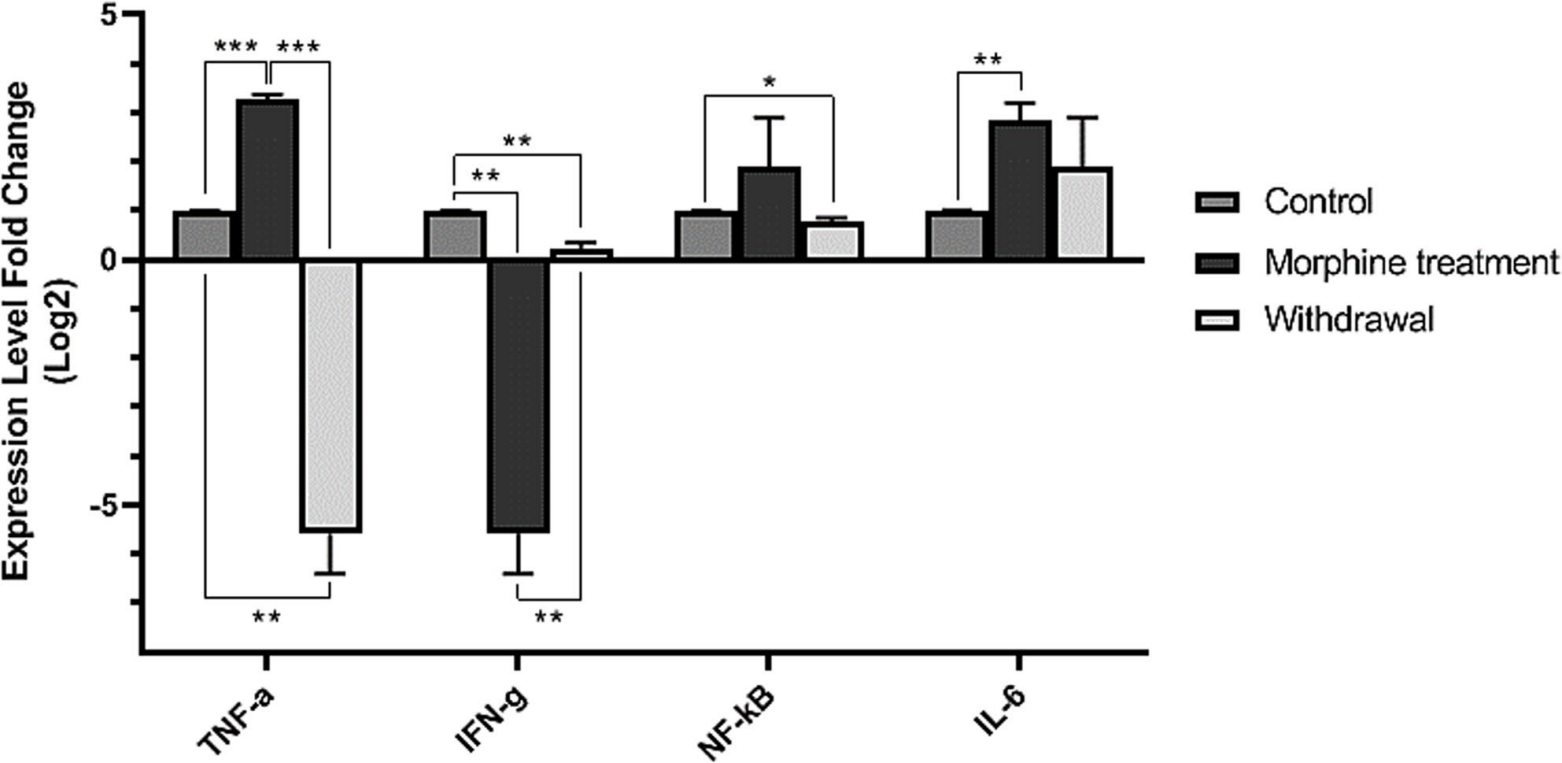
Expression levels of inflammatory genes by relative real-time PCR amplification from rats challenged by morphine abuse and withdrawal. The obtained data for each experimental group is reported as the mean ± SEM (*n* = 10). The distribution of the individual data in each group is also seen on each bar. ***: *P* < 0.001

The expression levels of *TNF-α*, *IFN-γ*, *IL-6*, and *NF-κB* in rat liver were measured by relative real-time PCR amplification (Fig. 3). Gene expression analysis revealed a significant upregulation in the mRNA levels of key pro-inflammatory mediators in the livers of morphine-treated rats compared to saline controls (*TNF-α*, *P* < 0.0001; *NF-κB*,* P =* 0.41; *IL-6*,* P =* 0.005). *IFN-γ* expression decreased significantly under morphine treatment (*P* < 0.001). Following the withdrawal period, mRNA expression of *TNF-α* (*P* < 0.01) and *IFN-γ* (*P* = 0.004) decreased relative to the treatment phase. In contrast, the transcriptional upregulation of *NF-κB* (*P* = 0.03) and *IL-6* (*P* = 0.4) persisted, indicating a sustained shift in the hepatic inflammatory gene profile even after morphine cessation. The levels of *TNF-α* (*P* = 0.0004) and *IFN-γ* (*P* = 0.002) exhibited significant differences in the morphine-treated group when compared to the group after morphine withdrawal. In contrast, the changes in *NF-κB* (*P* = 0.3) and *IL-6* (*P* = 0.4) expression levels did not exhibit significant differences (Table 4).

**Table 4:** Comparison of expression level of inflammatory genes between morphine-treated cases and controls*

Inflammatory Genes	Morphine-treated (Mean ± SEM)	Withdrawal (Mean ± SEM)
***TNF-a***	3.28 ± 0.08	-5.59 ± 0.82
***IFN-g***	-5.59 ± 0.82	0.2 ± 0.14
***NF-kB***	1.91 ± 0.99	0.77 ± 0.75
***IL-6***	2.86 ± 0.33	1.91 ± 0.99

*Data are presented as Mean ± SEM

**P-value < 0.05 was considered statistically significant

## Discussion

The current research examined how morphine dependence affects the gut-liver axis and inflammatory processes in a rat model, focusing on significant gut microbiota genera such as *Lactobacillus*, *Bifidobacterium*, *Bacteroides*, *Faecalibacterium*, and *Clostridium*. Our results indicate that morphine exposure notably changes the composition of gut microbiota, potentially leading to liver inflammation through disrupted cytokine signaling. Surprisingly, a study found a rapid and significant alteration in gut microbiota composition within a single day morphine treatment compared to placebo-treated controls [12]. Prior to morphine treatment (Day 0), *Lactobacillus* (*P* > 0.05) and *Bifidobacterium* (*P* > 0.05) were present at stable levels, reflecting their status as dominant beneficial bacteria in a healthy gut. However, after 5 5 (*P* = 0.011; *P* = 0.003) and 10 days (*P* = 0.014; *P* = 0.0009) of morphine treatment, both genera showed a significant decline, consistent with previous findings that opioids can suppress beneficial bacteria, possibly due to changes in gut motility, reduced mucus production, or direct antimicrobial effects [19, 20]. The reduction of these bacteria may weaken the gut barrier, promoting bacterial translocation and systemic inflammation, including the activation of liver immune responses [21]. Some recovery of *Lactobacillus* (*P* = 0.014) and *Bifidobacterium* (*P* = 0.0009) was noted after a 10-day withdrawal period, but levels remained below baseline, suggesting that dysbiosis induced by opioids can persist even after cessation, potentially leading to ongoing gut dysfunction [19].

In contrast, *Clostridium* was found at low levels on Day 0 (*P* > 0.05), aligning with its dual role as both a commensal and an opportunistic pathogen. Following morphine treatment, *Clostridium* levels increased (Day 5, *P* = 0.005; and 10, *P* = 0.0001), which is consistent with evidence that opioids can promote its overgrowth. Morphine is known to reduce gut motility and induce constipation, creating a stagnant, more anaerobic environment that favors the proliferation of strict anaerobes like *Clostridium* [20]. Furthermore, opioid-induced alterations in bile acid secretion and composition can disrupt the antimicrobial properties of bile acid, which normally help regulate the growth of certain bacterial populations, including *Clostridium* spp [22, 23]. The decline in *Lactobacillus* and *Bifidobacterium* may reduce competition, allowing *Clostridium* to proliferate [24]. After 10 days of withdrawal, *Clostridium* levels decreased (*P* = 0.0001) yet remained elevated compared to baseline. Given the established role of specific *Clostridium* species in impairing intestinal barrier function and promoting inflammation through toxin production [25], we hypothesize that this persistent overgrowth may be one factor contributing to the prolonged gut barrier dysfunction and low-grade systemic inflammation observed even after morphine cessation [19].

*Bacteroides* and *Faecalibacterium*, both involved in immune modulation, exhibited different responses to morphine [26]. *Bacteroides*, abundant on Day 0 (*P* > 0.05), showed a gradual decline during morphine treatment with incomplete recovery. A significant reduction in *Bacteroides* was observed after 5 (*P* > 0.05) and 10 days (*P* > 0.05) of exposure. Opioids may reduce goblet cell function, limiting mucin availability that *Bacteroides* require for colonization [27, 28]. Morphine may also disrupt the bile acid-mediated growth regulation of *Bacteroides* [29]. Although *Bacteroides* levels partially recovered after 10 days of withdrawal (*P* > 0.05), they remained below baseline, indicating a long-term disturbance in gut stability [19]. The reduced *Bacteroides* may hinder polysaccharide fermentation, leading to decreased short-chain fatty acid production and impaired gut barrier function [30]. *Faecalibacterium* was also abundant on Day 0 (*P* > 0.05) but showed severe depletion during morphine treatment (5-day, *P* = 0.0005; 10-day, *P* = 0.00009), with limited recovery post-withdrawal (*P* = 0.00009). The significant decline in *Faecalibacterium* abundance suggests that opioid-induced gut dysmotility may create unfavorable conditions for this anaerobic bacterium [19]. Given the importance of butyrate in suppressing inflammation, its reduction could contribute to the elevated pro-inflammatory cytokines observed in the liver [31, 32]. The loss of *Faecalibacterium* may also be related to the decline in *Bacteroides*, which provides substrates for its growth [33]. After 10 days of withdrawal, *Faecalibacterium* levels remained significantly lower than baseline, indicating a long-term reduction in butyrate production, which is crucial for gut barrier integrity and inflammation suppression [19]. This may explain the elevated hepatic inflammatory markers even after stopping morphine [12].

Moreover, the remarkable microbiota stability observed in various timelines in control groups serves two important purposes. First, it verifies that experimental procedures and sampling timelines didn’t artificially affect microbial populations. Second, it provides a reliable baseline confirming that any dysbiosis observed in treatment groups results specifically from the experimental intervention rather than procedural artifacts [34]. This consistent control data significantly enhances the validity of longitudinal microbiome comparisons in intervention studies [35].

This study demonstrates that repeated morphine exposure significantly alters the expression of hepatic inflammatory cytokines (*TNF-α*, *P* < 0.0001; *NF-κB*,* P =* 0.41; *IL-6*,* P =* 0.005; *IFN-γ*, *P* < 0.001), with distinct patterns during treatment and withdrawal. The liver, being a primary detoxification organ, is particularly vulnerable to inflammatory signals from the gut [36]. The increase in *TNF-α* (*P* < 0.01), *IL-6* (*P =* 0.4(, and *NF-κB* (*P* = 0.03), alongside the decrease in *IFN-γ* (*P* = 0.004), suggests a pro-inflammatory shift in liver immune responses that partially resolves upon morphine cessation but leaves residual inflammatory dysregulation. These results are consistent with previous studies indicating that morphine disrupts gut barrier function, leading to endotoxemia and hepatic immune activation [37]. The dramatic increase in *TNF-α* aligns with prior research linking opioids to inflammation and liver damage [38]. *IL-6* was also significantly elevated, reinforcing morphine’s role in promoting acute-phase responses in the liver [39, 40]. The activation of NF-κB suggests that morphine enhances the expression of pro-inflammatory genes, likely through Toll-like receptor signaling in response to gut-derived endotoxins [41]. Conversely, the reduction in *IFN-γ* indicates that morphine may impair immune responses, which could heighten susceptibility to infections [42, 43]. The activation of the TNF-α, NF-κB, and IL-6 suggests inflammation driven by endotoxemia, potentially due to gut barrier dysfunction [44]. The depletion of beneficial bacteria like *Lactobacillus* and *Bifidobacterium* may exacerbate this process, as these bacteria help suppress pro-inflammatory cytokine production [33]. The loss of butyrate production leads to unchecked NF-κB activation and the release of inflammatory bacterial metabolites [45].

The withdrawal period resulted in partial microbial balance restoration; however, inflammatory markers remained elevated, indicating ongoing liver dysfunction. Following morphine cessation, cytokine expression showed mixed recovery patterns. *TNF-α* level decreased significantly but may not have fully normalized, suggesting lingering low-grade inflammation. *NF-κB* levels remained elevated, indicating sustained activation of inflammatory pathways. *IL-6* did not show a significant decline, which could contribute to an increased risk of liver damages. The persistent activation of *NF-κB* and *IL-6* suggests ongoing low-grade inflammation, likely due to continued gut dysbiosis and endotoxin leakage [46, 47]. *IFN-γ* levels rebounded, indicating a partial restoration of Th1 immunity, suggesting that T-cell function may recover faster than innate immune responses [48]. This aligns with studies showing that probiotics can mitigate opioid-induced liver inflammation [21, 49]. The long-term consequences of opioid-induced dysbiosis highlight the need for therapeutic strategies aimed at restoring gut microbiota.

The present research supports the idea that modulating gut microbiota could be a potential strategy to alleviate morphine-induced liver inflammation. Repeated morphine administration fosters a pro-inflammatory environment in the liver, potentially accelerating liver injury and metabolic dysfunction [50]. While withdrawal reduces inflammation, it does not completely resolve it, emphasizing the need for microbiota-targeted and anti-inflammatory therapies [51]. Probiotic supplementation with *Lactobacillus* and *Bifidobacterium* has been shown to lower gut permeability and systemic inflammation in opioid-exposed models [52]. Furthermore, prebiotics that promote *Faecalibacterium* growth may help restore butyrate production, reducing inflammation [53]. Monitoring cytokines like TNF-α, IL-6, and NF-κB post-withdrawal could help identify patients at risk for persistent liver damage [54]. Future studies should investigate therapeutic interventions to expedite microbial recovery after opioid exposure and explore the role of bile acid metabolism in linking morphine, microbiota, and liver inflammation.

While our study provides strong molecular evidence for morphine-induced dysregulation of the gut-liver axis, it is important to acknowledge its limitations. The assessment of hepatic inflammation was based on the measurement of cytokine and transcription factor mRNA levels. Although this RT-qPCR data demonstrates a clear pro-inflammatory transcriptional shift, it does not constitute direct histological or protein-level confirmation of liver inflammation. Future investigations should build upon these findings by employing techniques such as histopathological examination (e.g., H&E staining for inflammatory cell infiltration), immunohistochemistry for cytokine localization within liver tissue, or western blot/ELISA to quantify protein expression. Such validation would solidify the link between the observed gut dysbiosis, the upregulation of inflammatory genes, and the manifestation of overt hepatic inflammatory pathology. Nonetheless, the persistent transcriptional changes reported here, particularly for *NF-κB* and *IL-6* post-withdrawal, provide a compelling rationale for these further studies.

## Conclusion

In conclusion, morphine dependence leads to rapid and sustained dysbiosis of gut microbiota, which persists even after cessation, characterized by a decline in beneficial bacteria and an increase in pro-inflammatory species. These changes likely contribute to liver inflammation through heightened cytokine production, highlighting the gut-liver axis’s role in opioid-related pathology. Restoring microbial balance may offer a new therapeutic approach to mitigate opioid-related liver damage, warranting further research into microbiota-targeted interventions into real-world treatments.

## Data Availability

Any relevant data supporting the findings of this study are accessible and can be provided directly by the authors.

## Abbreviations

MOP: mu opioid receptor
DOP: delta opioid receptor
KOP: kappa opioid receptor
NOP: nociceptin/orphanin opioid receptor
